# Fungal Bioleaching for Metal Recovery: Recent Progress and Technological Advances

**DOI:** 10.3390/jof12090635

**Published:** 2026-08-25

**Authors:** Angélica Ribeiro Soares, Xinran Xu, Marcela dos Passos Galluzzi Baltazar, Wen-Bing Yin

**Affiliations:** 1 State Key Laboratory of Microbial Diversity and Innovative Utilization, Institute of Microbiology, Chinese Academy of Sciences, Beijing 100101, China; angelsoares357@usp.br (A.R.S.);; 2 Chemical Engineering Department, University of São Paulo, Rua do Lago, 250–2º andar, São Paulo 05508-080, Brazil

**Keywords:** fungal bioleaching, metal recovery, secondary resources, organic acids, green metallurgy

## Abstract

Fungal bioleaching, as a green and sustainable technology for metal recovery, demonstrates unique advantages in addressing growing global metal demand and utilizing secondary resources. This review systematically summarizes recent progress and technological advances in fungal bioleaching for metal recovery. Fungi solubilize metals through three core mechanisms, acidolysis, complexolysis, and redoxolysis, mediated by organic acids (citric, oxalic, gluconic), while cell wall functional groups enable biosorption. Most frequently used genera in modern bioleaching (*Aspergillus*, *Penicillium*, *Trichoderma*) have achieved remarkable recoveries from e-waste (Cu, Li, Co up to 100%), rare earth elements (consortia up to 76%), tailings, and spent catalysts. Optimization of key parameters (pH, temperature, pulp density) and application of intelligent strategies (response surface methodology, machine learning) have further enhanced leaching efficiency. Strain engineering and synthetic biology offer new avenues for constructing hyper-efficient strains. Despite remaining bottlenecks such as slow kinetics and scale-up difficulties, fungal bioleaching holds broad industrial prospects for advancing the circular metal economy and the green metallurgical transition.

## 1. Introduction

Since the Industrial Revolution, global economic development has long relied on fossil fuel consumption, with coal, natural gas, and petroleum accounting for approximately 87% of global energy consumption in 2024. The ongoing global energy transition further amplifies the demand for strategic metallic minerals, as clean energy facilities such as wind turbines, photovoltaic systems, and electric vehicles are highly dependent on lithium, cobalt, nickel, and rare earth elements (REEs). Statistically, global annual consumption of metals and alloys reaches 2 billion tons, contributing 40% of industrial greenhouse gas emissions and consuming 10% of global energy resources, while primary mineral extraction requires 3.2 billion tons of raw minerals annually [1]. Large-scale primary mining inevitably results in the depletion of high-grade ore resources and produces substantial industrial waste. This creates a paradox in the development of green technologies, where efforts to reduce fossil fuel pollution through sustainable energy solutions inadvertently accelerate the consumption of metal resources and increase ecological pressure [2,3].

Two major secondary metal waste streams have become prominent global environmental and resource issues: mining tailings and Waste Electrical and Electronic Equipment (WEEE). Global mining activities produce billions of tons of tailings every year, which contain residual low-concentration valuable metals but pose severe environmental risks without proper disposal [4]. Meanwhile, the rapid iteration of electronic devices leads to explosive growth of e-waste, with a global annual output of 62 million tons and an annual growth rate of 3–5% [5]. E-waste is rich in high-value metals such as copper, gold, silver, lithium, and REEs, with metal concentrations far exceeding those of natural ores, making it a typical urban mine with high resource potential [6,7].

Traditional metal recovery technologies are dominated by pyrometallurgy and hydrometallurgy. Pyrometallurgy achieves metal separation through high-temperature smelting, which is energy-intensive, produces substantial greenhouse gas emissions and toxic flue gases, and is prone to slag entrapment, which can easily cause precious metal loss. Hydrometallurgy adopts strong acid and alkali chemical leaching, with problems of corrosive reagent consumption, large wastewater discharge, and secondary environmental pollution [8]. Both technologies are economically unviable for low-grade complex secondary waste and fail to meet the dual goals of resource recycling and green development.

**Figure 1:**
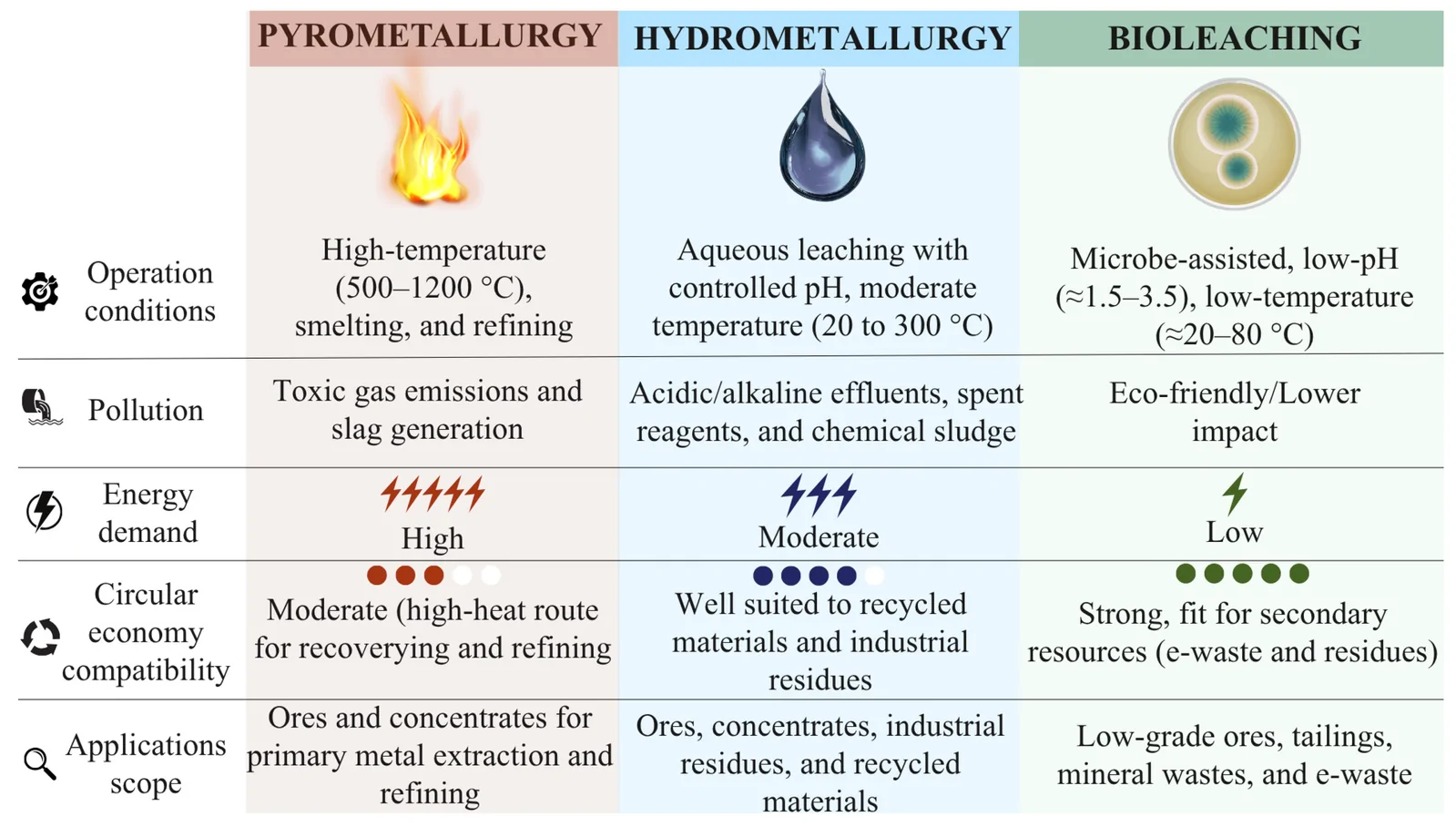
Comparison of traditional metallurgy and fungal bioleaching technology 2022.

Bioleaching, a microbial-mediated metal solubilization technology, has emerged as a sustainable alternative to traditional metallurgy (Figure 1). Bacterial bioleaching has been widely applied in copper and gold extraction from primary ores, but it is limited by narrow pH adaptability and poor tolerance to complex waste matrices [9]. In contrast, fungal bioleaching exhibits unparalleled advantages: filamentous fungi are heterotrophic microorganisms that can grow over a wide pH range (pH 3.0–7.0), tolerate high concentrations of heavy metals, and utilize low-cost agricultural and industrial residues as carbon sources [10]. Fungi secrete abundant organic acids, including citric acid, oxalic acid, and gluconic acid, which efficiently dissolve and chelate metals from complex waste substrates such as tailings and e-waste, enabling green, low-cost metal recovery.

Despite the growing number of fungal bioleaching studies in recent years, existing reviews mostly focus on individual waste types or single leaching mechanisms, lacking systematic collation of fungal taxonomic and physiological characteristics, multi-factor synergistic regulation, and cutting-edge technological upgrades. There is also insufficient discussion of the bottlenecks in industrialization and the future industrial applications of fungal bioleaching. The recent literature highlights a rise not only in fungal bioleaching quantities but also a shift in research focus. Earlier research mainly centered on strain selection, leaching mechanisms, and proof-of-concept metal recovery. In contrast, recent studies increasingly prioritize process optimization, hybrid approaches, and molecular techniques such as transcriptomics, CRISPR, and synthetic biology. Additionally, more attention is being directed toward complex secondary sources, metal selectivity, process stability, and scale-up, reflecting a move toward more engineered and application-oriented bioleaching systems.

Against this background, this review comprehensively analyzes the latest progress in fungal bioleaching from 2020 to 2026, systematically elaborates on the core metabolic and leaching mechanisms, summarizes the performance of dominant fungal strains and key influencing factors, and sorts out the application status across various secondary metal resources. Advanced optimization strategies based on synthetic biology and composite synergistic technologies are highlighted, and the challenges and future research directions for industrial scaling are prospected. This review aims to provide theoretical and technical support for the construction of efficient, sustainable fungal bioleaching systems and promote the closed-loop recycling of critical metals in the circular economy.

## 2. Mechanisms of Fungal Bioleaching

Fungal bioleaching is a complex process primarily driven by the secretion of organic acids. Unlike autotrophic bacteria, heterotrophic fungi rely on carbohydrate metabolism to produce a variety of low-molecular-weight organic acids, which are the main agents responsible for metal solubilization. This process is governed by acidolysis and complexolysis, while redoxolysis may contribute in specific mineral systems involving redox-sensitive metals [11,12]. Furthermore, fungal biomass can interact with dissolved metal ions through biosorption, and intracellular metal-tolerance mechanisms help maintain fungal activity under metal stress [13]. Together, this shapes the overall efficiency of fungal bioleaching.

Organic acid metabolism is central to fungal bioleaching, with gluconic, oxalic, citric, and malic acids among the most relevant metabolites. Their relative production matters because each acid can influence metal dissolution, complexation, and subsequent recovery differently. *Aspergillus niger* mainly produces gluconic acid via extracellular glucose oxidation: glucose is liberated from sucrose and further oxidized by cell wall-bound glucose oxidase to generate glucono-δ-lactone, which spontaneously converts to gluconic acid [14]. This pathway is oxygen-dependent and can be efficiently enhanced under high-glucose conditions, with a maximum yield of 8205 mg/L reported in PCB leaching experiments [15]. Oxalic acid is the dominant metabolite of *Penicillium simplicissimum*, with reported yields of up to 9010 mg/L, and it exhibits strong metal-chelating and redox activity [15,16]. Citric acid and malic acid are intermediate products of the fungal tricarboxylic acid (TCA) cycle and play auxiliary roles in proton supply and metal complexation [10,12,17]. Fungal species and strains, carbon sources, and waste-substrate properties jointly determine organic acid types and yields, resulting in significant differences in leaching performance.

Oxalic acid is relevant for the recovery of rare-earth elements, as oxalate can induce the precipitation of these elements in the form of soluble rare-earth oxalates [18]. Consequently, depending on its concentration and solution composition, oxalic acid may participate in metal solubilization and downstream selective separation processes. For instance, leachates from NdFeB magnets have demonstrated the selective precipitation of rare-earth elements using oxalic acid, although excessive oxalate addition increases the risk of iron co-precipitation [19,20]. Therefore, the profile of fungal organic acids, rather than total acid production alone, is a critical parameter in designing selective bioleaching and metal recovery processes.

**Figure 2:**
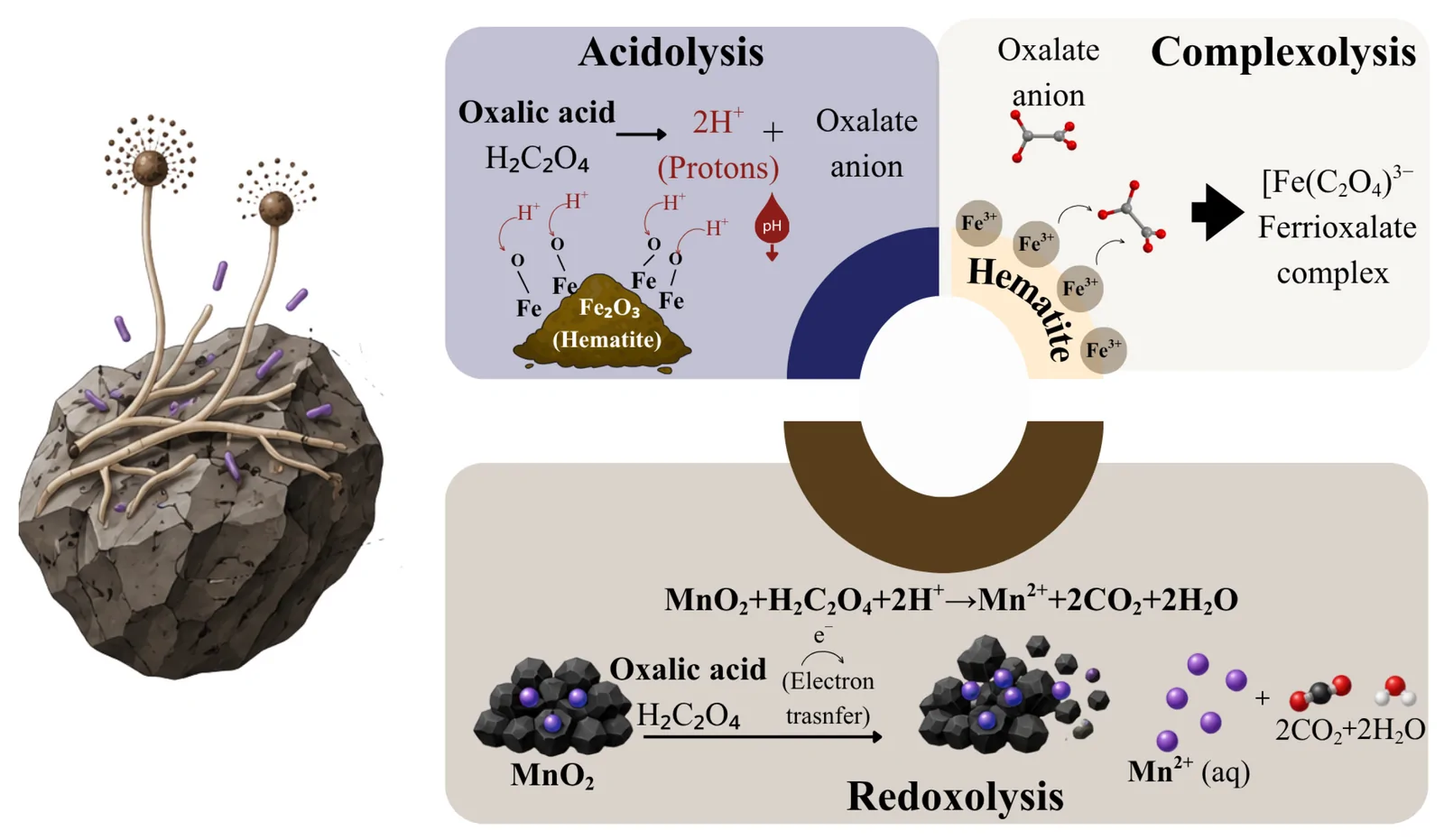
Schematic illustration of three core mechanisms of fungal bioleaching. Organic acids produced by fungi promote metal mobilization through acidolysis (Blue), by releasing H^+^ and lowering pH; complexolysis (yellow), through the formation of soluble metal-organic complexes; and redoxolysis (brown), through electron-transfer reactions that change metal oxidation states and enhance dissolution. Arrows indicate the direction of the reactions.

Acidolysis, complexolysis, and, in specific Cases, redoxolysis constitute the main metal-solubilization pathways in fungal bioleaching (Figure 2). Acidolysis is the most fundamental mechanism, in which organic acids release protons that lower the environmental pH and promote the dissolution of metal-bearing minerals through proton attack on mineral surfaces [21,22]. Complexolysis occurs when organic acid anions, particularly oxalate and citrate, form soluble complexes with metal ions, enhancing metal mobilization and preventing precipitation in the leaching systems [23,24]. Redoxolysis is driven by biologically mediated oxidation–reduction reactions in which electron transfer alters metal oxidation states, potentially destabilizing the solid phase and enhancing solubility. In fungal systems, this may occur through redox-active metabolites such as oxalic acid, which reduces Mn(IV) to Mn(II) [25], or through oxidoreductases such as laccase (Lac), manganese peroxidase (MnP), and lignin peroxidase (LiP), which may contribute to the transformation of metal-bearing matrices [26]. However, their contribution to metal leaching should not be generalized. In *Phaenorochaete chrysosporium*-mediated copper leaching, the activities of laccase, manganese peroxidase, and lignin peroxidase showed only weak and statistically insignificant correlations with Cu leaching efficiency (r = 0.45–0.46, *p* > 0.05). Additionally, heavy-metal exposure inhibited enzyme production [26]. Thus, these enzymes might assist in matrix degradation or indirectly promote metal mobilization, but current evidence does not support a direct enzymatic regulation of copper leaching.

While acidolysis, complexolysis, and redoxolysis are the primary mechanisms responsible for metal solubilization during fungal bioleaching, fungal biomass can also interact with dissolved metal ions through biosorption. Functional groups present in the cell wall, including hydroxyl, carboxyl, amino, and phosphoryl moieties, can bind metal ions through ion exchange, electrostatic interactions, and coordination reactions [27]. *A. niger*, *A. flavus*, and *P. simplicissimum* proved to be effective at adsorbing dissolved metal ions from complex battery leachates, highlighting the potential of fungal biosorption as an environmentally friendly post-leaching purification or polishing strategy [11].

In addition, fungi have evolved complete molecular regulation mechanisms to adapt to high-metal environments. Metal tolerance genes and specific metal transport proteins can regulate intracellular metal ion concentrations, mitigate heavy metal toxicity in cells, and maintain normal metabolic activity of fungi in complex, high-metal waste matrices [13,28].

## 3. Dominant Fungal Genera and Operational Characteristics

Based on fungal genera identified in the literature compiled for this review, *Aspergillus* was the most frequently represented genus (51.7%), followed by *Penicillium* (15.5%) and *Trichoderma* (5.2%). However, these three genera do not encompass the full fungal diversity reported for metal bioleaching. Other fungal genera collectively represented 27.6% of the occurrences and included *Phanerochaete* [29], *Lentinus*, *Pleurotus*, *Ganoderma*, *Trametes* [30], *Purpureocillium* [31], *Talaromyces* [32], *Paraconiothyrium*, *Paraphaeosphaeria*, *Phoma* [33], among others. This distribution highlights the broader taxonomic diversity of fungi investigated for metal mobilization and recovery. Different fungal genera, species, and strains exhibit distinct metabolic and leaching characteristics, offering unique application advantages across different secondary waste systems.

*A*. *niger* is one of the most frequently used fungal species in bioleaching studies and exhibits high metal-leaching performance, with the strongest comprehensive metabolic capacity. It can simultaneously secrete gluconic acid, oxalic acid, and citric acid, adapting to acidic, neutral, and weakly alkaline environments. Under sucrose culture conditions, *A. niger* can achieve 100% leaching efficiency for zinc and nickel in waste mobile phone PCBs, and 94% for copper [15]. It also shows excellent adaptability to low-grade tailings, spent catalysts, and LCD waste powder, with good recovery of precious metals and REEs [12]. *P*. *simplicissimum* is known for its high oxalic acid yield and for not secreting gluconic acid under conventional culture conditions. Its strong redox and chelating ability endows it with excellent performance in copper, nickel, and zinc leaching, with copper leaching efficiency up to 100% in high-pulp-density PCB systems [16]. *Trichoderma* is primarily used for leaching low-grade mineral waste, offering unique advantages for the recovery of Nickel and zinc from tailings samples [34].

**Figure 3:**
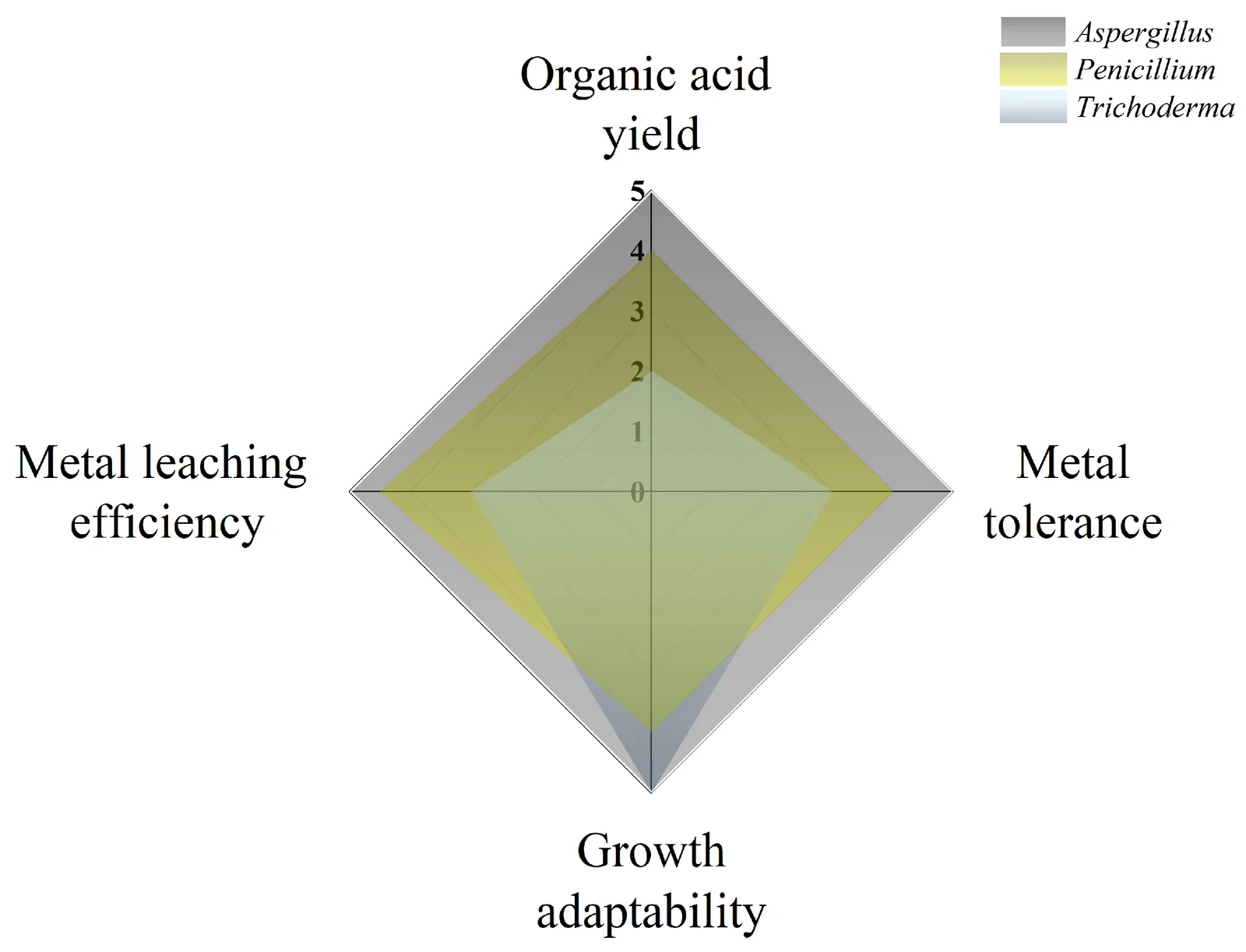
Performance comparison of the main fungi used in bioleaching. Radar chart showing the relative performance of *Aspergillus*, *Penicillium*, and *Trichoderma* in organic acid production, metal tolerance, growth adaptability, and metal leaching efficiency. Scores, ranging from 1 (very low) to 5 (very high), are based on literature reports and consider organic acid diversity, metal tolerance range, environmental adaptability, and overall bioleaching performance across multiple studies.

The physiological characteristics of fungal strains directly determine bioleaching performance. Excellent bioleaching fungi have three core traits: rapid vegetative growth, efficient secretion of organic acids, and strong tolerance to metal stress (Figure 3). Wild-type strains isolated from metal-contaminated soil and mining areas usually exhibit greater natural resistance to metals, whereas laboratory-domesticated and mutated strains show higher organic acid yields and leaching efficiency [35,36]. Strain screening and domestication are key preconditions for high-efficiency bioleaching: common strategies include directional domestication via metal-stress gradients, ultraviolet mutagenesis, and adaptive culture with low-cost substrates, which can significantly improve strains’ adaptability to complex waste matrices.

There are significant performance differences between wild indigenous strains and modified strains. Indigenous strains exhibit strong environmental adaptability and stable metabolic activity in real-world waste systems, but their organic acid yields and leaching efficiencies are relatively low. Genetically modified strains obtained via synthetic biology and mutation technology can realize targeted enhancement of organic acid synthesis pathways and metal tolerance. For example, CRISPR-modified yeast strains can achieve ultra-high oxalic acid production, realizing 99% precipitation and recovery of rare earth elements [37]. Modified fungal strains exhibit higher leaching efficiency and process stability, indicating greater potential for industrial applications.

## 4. Influencing Factors of Fungal Bioleaching Performance

Fungal bioleaching can be performed using three main operational approaches: one-step, two-step, and spent medium systems. In the one-step approach, fungal inoculation and substrate addition occur simultaneously, allowing fungal growth, metabolite production, and metal solubilization to proceed concurrently in a single reactor. This configuration is considered the simplest and most economical option due to reduced processing steps and equipment requirements [38]. In the two-step approach, the fungus is first cultivated separately until it reaches the exponential growth phase or produces enough bioleaching metabolites, after which the solid substrate is introduced [39]. The spent medium approach involves an additional separation step in which the fungal biomass is removed after cultivation, and only the metabolite-rich culture filtrate is contacted with the solid substrate. This strategy eliminates direct interactions between the mineral substrate and the fungal cells while maintaining the leaching activity of extracellular metabolites [40].

The fungal bioleaching efficiency is affected by multi-dimensional synergistic factors including environmental parameters, nutritional conditions, waste substrate properties, and biological characteristics. Reasonable parameter optimization can significantly improve organic acid secretion, fungal metabolic activity, and metal solubilization efficiency, thereby enabling high-efficiency bioleaching [41].

Environmental parameters are the most direct regulatory factors for fungal metabolic activity (Table 1). The pH value determines fungal growth status and the organic acid synthesis pathway: most fungi grow optimally at pH 3.0–6.0, and changes in system pH directly affect proton concentration and the equilibrium of metal-chelating reactions. Temperature affects fungal growth rate and enzyme activity: the optimal growth temperature for most mesophilic bioleaching fungi is 20–35 °C. Notably, the spent medium leaching system without constraints from living fungal cells can operate at a high temperature of 60–70 °C to accelerate metal dissolution kinetics and improve recovery efficiency. In addition, shaking speed and dissolved oxygen content regulate fungal aerobic metabolism: sufficient oxygen supply is essential for gluconic acid synthesis and cell proliferation. At the same time, appropriate shaking can increase the contact area between fungal metabolites and waste substrates, thereby reducing mass-transfer resistance.

Nutritional conditions determine the level of fungal organic acid metabolism. Carbon source type and concentration are core factors affecting acid production: sucrose and glucose are the most efficient carbon sources for bioleaching fungi and can be readily converted into various organic acid precursors. Different carbon source concentrations significantly regulate acid yield: high-concentration sucrose (100 g/L) maximizes gluconic acid yield in *A. niger*, whereas medium- and low-concentration glucose is more suitable for oxalic acid accumulation [15]. Nitrogen source type and C/N ratio affect fungal growth rate and metabolic flow distribution: an appropriate nitrogen supply promotes mycelial growth, whereas low-nitrogen stress redirects metabolic flow toward organic acid synthesis, thereby improving leaching performance.

Solid waste substrate properties determine the difficulty of metal solubilization. Particle-size refinement can increase the specific surface area of waste materials, enhance contact with fungal metabolites, and significantly improve leaching efficiency. Pulp density is a key parameter that balances processing capacity and leaching efficiency: the optimal pulp density in current laboratory studies ranges from 1–16 g/L or 1–8% (*w*/*v*). Excessively high pulp density will lead to excessive accumulation of toxic metals, inhibit fungal metabolic activity, and reduce recovery rate, while low pulp density results in low processing efficiency [48]. In addition, the impurity composition of waste substrates (such as quartz, silicate, and brominated flame retardants in e-waste) will interfere with organic acid reactions and fungal growth, increasing the difficulty of bioleaching.

Biological factors include inoculum size and incubation time. Appropriate inoculum dosage can shorten the fungal growth cycle and accelerate metabolite accumulation; insufficient inoculum leads to slow strain proliferation and delayed acid production, while excessive inoculum causes nutrient competition and metabolic waste. The incubation cycle is usually 7–15 days: the early stage is the fungal growth and metabolite accumulation stage, and the middle and late stages are the main metal solubilization stages. Reasonable control of incubation time can avoid metal reprecipitation and improve resource recovery efficiency [44].

## 5. Recent Application Progress in Diverse Secondary Resources

**Figure 4:**
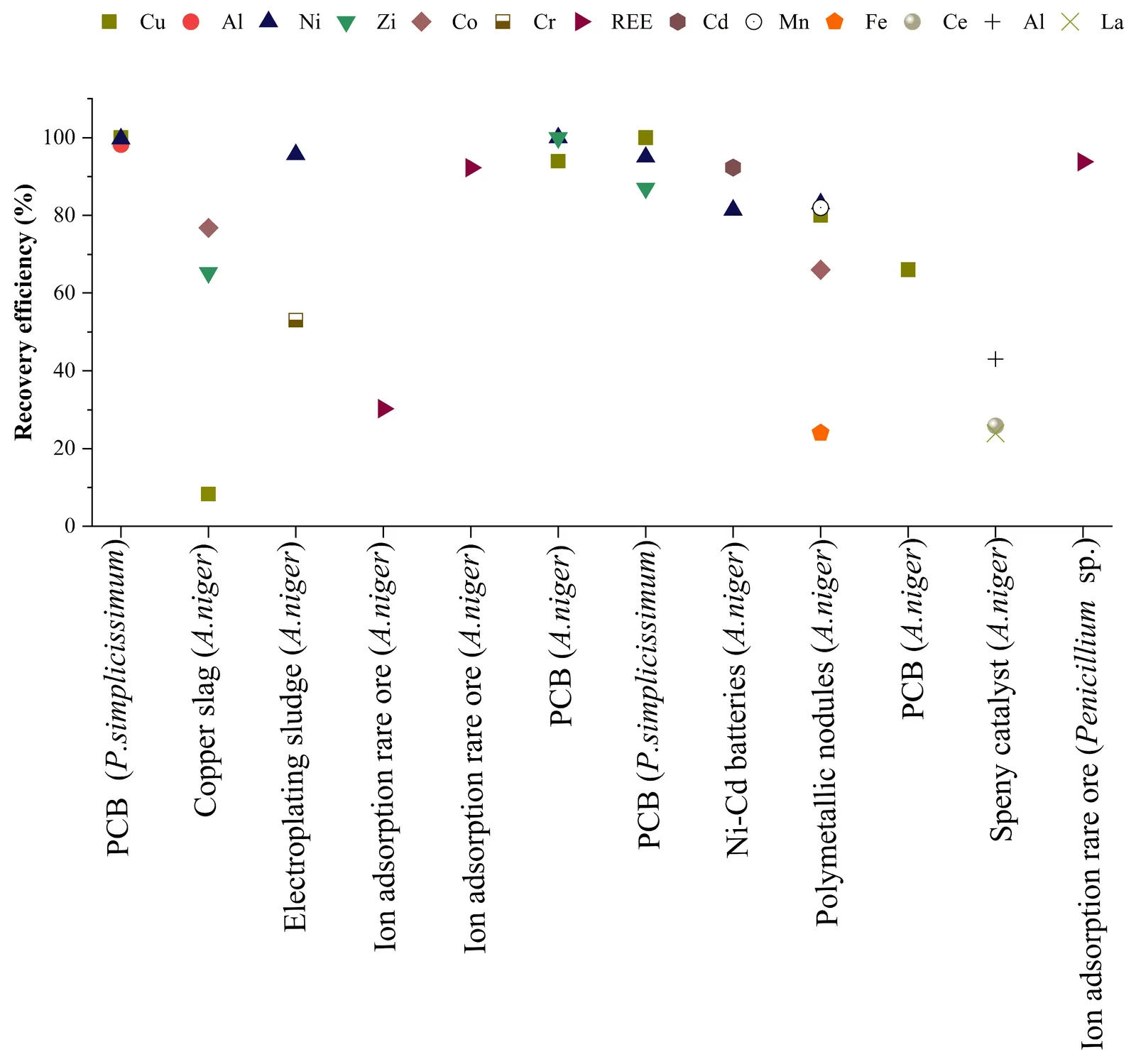
Application scenarios and metal recovery efficiency of fungal bioleaching for secondary resources.

Fungal bioleaching has been applied in metal recovery from various secondary resources, covering e-waste, mining tailings, metallurgical residues, and spent catalysts (Figure 4). Among them, e-waste is the most studied substrate, accounting for 60% of all fungal bioleaching research, whereas mineral waste and other industrial residues remain in the exploratory stage [43]. Different secondary resources have distinct matrix characteristics and metal compositions, leading to varying bioleaching performance and development potential.

E-waste, such as printed circuit boards (PCBs), mobile phone components, and waste lithium batteries) is the most mature application scenario for fungal bioleaching. E-waste has ultra-high metal concentrations: the copper content of waste smartphone PCBs ranges from 16–82 wt%, and the concentrations of gold and silver are 30–60 times higher than those in natural ores [49]. Fungal bioleaching can efficiently recover base metals (Cu, Zn, Ni, Al), precious metals (Au, Ag, Pd), and REEs from e-waste. *A. niger*-mediated one-step bioleaching can achieve 100% recovery of Zn and Ni and 94% recovery of Cu from waste mobile phone PCBs [15]. The spent medium system of *A. niger* PTCC 5010 can recover 57% of Pd and 33% of Pt from waste circuit boards, realizing efficient recovery of precious metals [45]. For waste lithium-ion batteries, fungal organic acids can efficiently dissolve lithium, cobalt, and nickel, providing a green path for battery metal recycling.

Mining tailings and metallurgical residues are the largest-volume secondary metal resources. Global annual tailings production exceeds 13 billion tons and contains low concentrations of nickel, cobalt, manganese, REEs, and other strategic metals that cannot be recovered using traditional technologies [4]. Fungal bioleaching offers unique advantages for the treatment of low-grade tailings: *Trametes versicolor* OG2 can recover 41.82% nickel from olivine tailings and 30.97% from serpentine tailings [43]. In addition, fungal bioleaching can also treat metallurgical slags and red mud, recovering aluminum, vanadium, and other metals while reducing the environmental risk of solid waste accumulation [50].

But there are additional challenges, including limited selectivity and the formation of secondary precipitates that hinder downstream metal recovery [36] and the heterogeneous composition of ores and waste streams. Feedstocks might include silicate-rich, alkaline, or refractory phases that restrict metal accessibility and require pretreatment before bioleaching, such as calcination to break the strong bond between silica and metals [51]. Therefore, fungal bioleaching relies not just on microbial efficiency but also on a comprehensive understanding of the mineralogical and chemical properties of the material being processed. Because performance is sensitive to feedstock variability and operating conditions, overcoming the current bottleneck will require advances in strain development, process optimization, and closer integration of microbiological, metallurgical, and engineering principles.

Industrial fly ash and spent catalysts are emerging high-value secondary resources. Industrial fly ash contains enriched aluminum, iron, and rare earth elements, while spent industrial catalysts are rich in platinum, palladium, and other precious metals. Fungal bioleaching can extract precious metals from spent automobile catalysts and electroplating sludge: *A. niger* can produce high concentrations of oxalic acid to dissolve chromium, nickel, and other metals in electroplating sludge, achieving a nickel recovery rate up to 95.7% [10]. This technology provides a low-cost, green recovery method for recycling high-value metals from industrial solid waste.

In terms of rare earth and heavy metal recycling, fungal bioleaching has made remarkable progress. Fungal organic acids can dissolve light and heavy REEs in fluorescent lamp waste and tailings, with the recovery rate of yttrium and lutetium exceeding 100% in some optimized systems [47]. For toxic heavy metals such as chromium, cadmium, and lead in contaminated waste, fungal bioleaching can realize resource recovery while detoxifying waste substrates, achieving dual benefits of environmental remediation and resource utilization [46].

## 6. Advanced Optimization Strategies and Technological Upgrades

**Figure 5:**
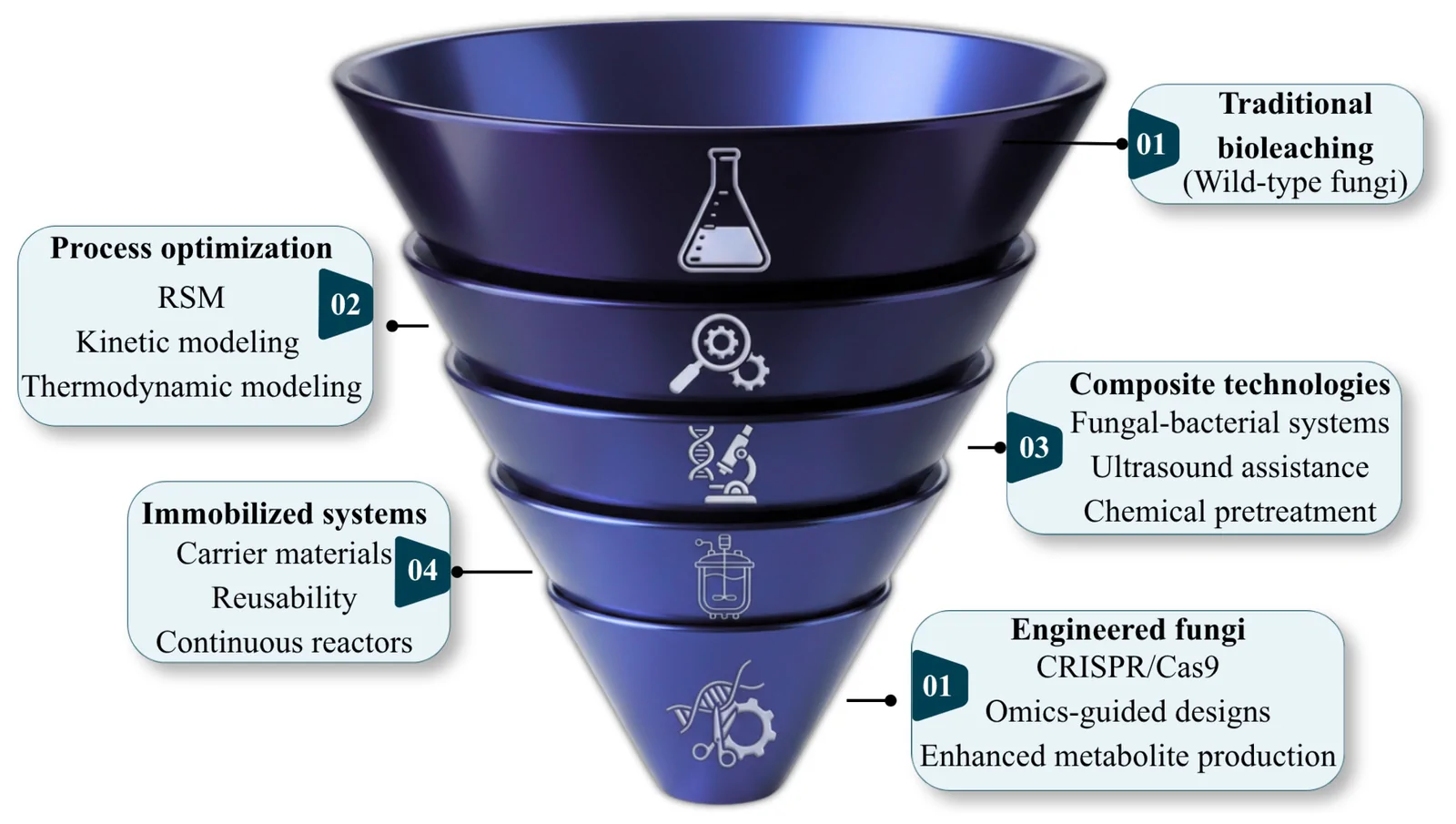
Technical upgrade roadmap and industrialization framework of fungal bioleaching.

To solve the problems of low leaching kinetics, unstable efficiency, and poor substrate adaptability of traditional fungal bioleaching, a variety of advanced optimization strategies have been developed in recent zs, including process parameter modeling optimization, multi-technology synergistic composite leaching, immobilized cell technology, and synthetic biology strain modification, which greatly improve the efficiency and industrial applicability of fungal bioleaching (Figure 5) [52].

Process parameter modeling and optimization are the most mature and convenient approaches. Response surface methodology (RSM) is widely used to construct multi-factor interaction models of pH, temperature, pulp density, and incubation time to predict the optimal parameter combination and maximize metal recovery efficiency. Kinetic and thermodynamic modeling can clarify the rate-limiting step of the bioleaching reaction, reveal the synergistic mechanism of multi-parameter regulation, and provide theoretical guidance for process amplification [53]. Compared with single-factor optimization, model-based multi-dimensional optimization can increase metal leaching efficiency by 10–30% and significantly shorten the leaching cycle.

Multi-technology synergistic composite leaching overcomes the limitations of single-fungal leaching. Fungal-bacterial consortium bioleaching combines the advantages of fungal organic acid leaching and bacterial redox leaching, thereby achieving synergistic enhancement of acidolysis and redoxolysis and significantly improving the leaching efficiency of refractory metals. Ultrasound-assisted bioleaching uses ultrasonic vibration to break fungal mycelial and waste particle agglomeration, increase mass transfer efficiency, and accelerate organic acid–metal reaction. Chemical-fungal combined leaching employs mild chemical pretreatment to disrupt the complex structure of waste substrates, remove inhibitory impurities, and enhance the accessibility of metals to fungal metabolites [54]. These synergistic technologies complement the limitations of single-fungal bioleaching and expand the range of applicable substrates.

Immobilized fungal cell technology addresses the problems of poor fungal recyclability and difficult solid–liquid separation in traditional suspended-culture systems. By immobilizing fungal mycelium on carrier materials such as activated carbon and alginate, the stability of fungal cells can be improved, repeated utilization of strains can be realized, and the cost of strain culture can be reduced. Immobilized fungi maintain stable organic acid secretion in continuous leaching systems, thereby solving the problem of low efficiency caused by strain loss in scale-up experiments and providing technical support for continuous industrial production.

Synthetic biology and genetic engineering modification are the core cutting-edge technologies for long-term performance improvement of fungal strains. With the development of CRISPR/Cas9 genome editing and multi-omics technologies, targeted modification of fungal metabolic pathways can be achieved: redirecting metabolic flux toward organic acid synthesis, enhancing the expression of glucose oxidase and oxalic acid synthetase, and significantly increasing organic acid yield. Genetic modification can also enhance fungal metal tolerance and stress resistance, enabling strains to maintain high metabolic activity in high-pulp-density and high-toxicity waste systems [37]. Synthetic biology-based modifications fundamentally optimize the intrinsic performance of fungal strains and overcome the performance bottlenecks of wild-type strains.

## 7. Challenges, Future Perspectives and Industrial Prospects

Although fungal bioleaching offers significant environmental advantages and excellent laboratory-scale metal recovery performance, multiple technical, economic, and industrial bottlenecks still hinder its large-scale industrial adoption. Clarifying existing challenges and future research directions is crucial for the industrial transformation of this technology.

The primary technical bottleneck is slow leaching kinetics and a long reaction cycle. Fungal bioleaching usually requires 7–15 days to achieve stable metal recovery, while traditional chemical leaching can complete the reaction within several hours, resulting in low industrial production efficiency [22]. In addition, fungal metabolic activity is sensitive to fluctuations in waste substrates: complex impurities and high-concentration toxic metals in actual industrial waste will inhibit fungal growth and organic acid secretion, leading to unstable leaching efficiency. The low selectivity of fungal metabolites often leads to simultaneous leaching of impurity metals, thereby increasing the difficulty of subsequent metal separation and purification [36].

Economic and environmental challenges cannot be ignored. Traditional fungal culture relies on high-purity sucrose and glucose media, and their high cost limits the economic viability of industrial applications. The waste fungal biomass generated after leaching contains enriched heavy metals, requiring safe disposal and treatment, thereby increasing operating costs. At present, most studies are limited to small-scale laboratory batch experiments, and there is a lack of continuous amplification and pilot-scale test data, making it difficult to verify the stability and economic feasibility of large-scale production [55].

Future research directions of fungal bioleaching focus on four core aspects: first, intelligent strain modification based on synthetic biology, to breed high-yield acid, high-metal-tolerance, and high-selectivity engineered fungal strains; second, development of low-cost culture media using agricultural and industrial waste residues to reduce production costs and realize waste resource coupling utilization; third, construction of intelligent process control systems, combining machine learning and kinetic models to realize real-time parameter regulation and stable efficient leaching; fourth, development of new composite pretreatment and synergistic leaching technologies to improve the adaptability of fungal bioleaching to complex refractory waste [56].

In terms of industrialization prospects, fungal bioleaching is best suited to low-grade, complex secondary wastes that are uneconomical to treat using traditional metallurgical technologies. With the continued improvement of carbon-emission policies and circular-economy requirements, the environmental advantages of fungal bioleaching will gradually offset its economic disadvantages. Future industrial promotion can adopt a step-by-step scaling path: starting with laboratory optimization and pilot-scale continuous testing, gradually advancing to industrial applications in e-waste and low-grade tailings treatment, and ultimately establishing a complete set of green metal recovery technology systems to support the sustainable development of the metal industry and the global energy transition [57].

## 8. Conclusions

Fungal bioleaching, as an emerging green biohydrometallurgical technology, provides a sustainable solution for the resource recycling of critical metals from secondary wastes including e-waste and mining tailings. Compared with traditional pyrometallurgy and hydrometallurgy, it offers notable advantages, including low energy consumption, low carbon emissions, mild reaction conditions, and strong adaptability to complex substrates, aligning with the development trend of the circular metal economy and global carbon neutrality goals.

This review systematically summarizes the core research progress in fungal bioleaching: it relies on the synergistic effects of organic acid-mediated acidolysis, complexolysis, and redoxolysis for metal solubilization, followed by fungal biosorption processes that contribute to metal immobilization, enrichment, and recovery. Frequently used fungal genera, particularly *Aspergillus* and *Penicillium*, show excellent leaching performance in various secondary waste systems, and environmental parameters, nutritional conditions, and substrate properties precisely regulate their efficiency. Advanced optimization technologies, including process modeling, multi-technology synergy, immobilized cells, and synthetic biology-based modifications, have overcome the performance limitations of traditional fungal bioleaching.

At present, the industrialization of fungal bioleaching is still restricted by slow leaching kinetics, unstable scale-up performance, and high medium costs. Future research should focus on intelligent strain modification, low-cost medium development, and intelligent process optimization to improve the technology’s efficiency, stability, and economy. With continuous technological iteration and industrial verification, fungal bioleaching is expected to become a key supporting technology for secondary metal resource recycling, alleviate global pressure on critical metal supply, reduce the ecological and environmental damage caused by primary mining, and promote the sustainable and high-quality development of the metallurgical industry.

## Figures and Tables

**Table 1:** Fungal bioleaching efficiencies under different operational conditions.

Approaches	Fungi	Target Metals	Extraction (%)	Time (Days)	Pulp Density	Temperature	pH	Reference
One-step	*Aspergillus niger*	Cu	66	10	8% (*w*/*v*)	35	2	[42]
Two-step	*Trametes versicolor* OG2	Ni	41.82 (Olivine) 30.97 (Serpentine)	7	2% (*w*/*v*)	20	5	[43]
One-step	*Aspergillus niger*	Zn	100	15	4 g/L	30	10	[15]
		Ni	100					
		Cu	94					
	*Penicillium simplicissimum*	Zn	87					
		Ni	95					
		Cu	100					
One-step	*Aspergillus nidulans*	U	80	7	3% (*w*/*v*)	30	3	[44]
Spent medium	*Aspergillus niger* PTCC 5010	Pt	33	1	5 g/L	30	-	[45]
		Pd	57					
One-step	*Aspergillus niger*	Mn	82	11	2.5% (*w*/*v*)	30	pH 3	[41]
		Cu	80					
		Ni	83					
		Co	66					
		Fe	24					
Spent medium	*Aspergillus tubingensis* FMS1	Ca	58	28	1% (*w*/*v*)	30	-	[46]
		Co	53					
		Ni	52					
One-step	*Penicillium simplicissimum* BBRC20019	Cu	100	-	16 g/L	30	pH 9	[16]
		Al	98					
		Ni	70					
Spent medium	*Aspergillus niger* PTCC 5010	Cr	53	1	10 g/L	66	-	[10]
		Ni	95.7	1				
Spent medium	*Aspergillus niger* PTCC 5010	Al	81.4	1 d 5 h	10 g/L	70	-	[12]
		As	69.1					
		In	60					
		Sr	33.3					
Spent medium	*Aspergillus niger* (CICC 40349)	REEs	30.25	7	Liquid/solid ratio—2:1	-	~2.69–3.21	[47]
		Y	>100					
		Lu	>100					

## Data Availability

The original contributions presented in this study are included in the article. For further inquiries, contact the corresponding authors.
